# Perinatal mental health and COVID-19: Navigating a way forward

**DOI:** 10.1177/00048674221137819

**Published:** 2022-11-28

**Authors:** Katharine A Smith, Louise M Howard, Simone N Vigod, Armando D’Agostino, Andrea Cipriani

**Affiliations:** 1 Department of Psychiatry, University of Oxford, Oxford, UK; 2 Oxford Health NHS Foundation Trust, Oxford, UK; 3 Oxford Precision Psychiatry Lab, NIHR Oxford Health Biomedical Research Centre, Oxford, UK; 4 Section of Women’s Mental Health, Health Service and Population Research Department, Institute of Psychiatry, Psychology and Neuroscience, King’s College London, London, UK; 5 Women’s College Hospital and Women’s College Research Institute, Toronto, ON, Canada; 6 Department of Psychiatry, Faculty of Medicine, University of Toronto, Toronto, ON, Canada; 7 Department of Health Sciences, Università degli Studi di Milano, Milano, Italy

**Keywords:** Pregnancy, COVID-19, perinatal mental health, telepsychiatry

## Abstract

The COVID-19 pandemic and its aftermath have increased pre-existing inequalities and risk factors for mental disorders in general, but perinatal mental disorders are of particular concern. They are already underdiagnosed and undertreated, and this has been magnified by the pandemic. Access to services (both psychiatric and obstetric) has been reduced, and in-person contact has been restricted because of the increased risks. Rates of perinatal anxiety and depressive symptoms have increased. In the face of these challenges, clear guidance in perinatal mental health is needed for patients and clinicians. However, a systematic search of the available resources showed only a small amount of guidance from a few countries, with a focus on the acute phase of the pandemic rather than the challenges of new variants and variable rates of infection. Telepsychiatry offers advantages during times of restricted social contact and also as an additional route for accessing a wide range of digital technologies. While there is a strong evidence base for general telepsychiatry, the particular issues in perinatal mental health need further examination. Clinicians will need expertise and training to navigate a hybrid model, flexibly combining in person and remote assessments according to risk, clinical need and individual patient preferences. There are also wider issues of care planning in the context of varying infection rates, restrictions and vaccination access in different countries. Clinicians will need to focus on prevention, treatment, risk assessment and symptom monitoring, but there will also need to be an urgent and coordinated focus on guidance and planning across all organisations involved in perinatal mental health care.

The COVID-19 pandemic has evolved rapidly and had global impacts on all areas of our lives. Although restrictions have been lifted in many countries, case rates have continued to fluctuate with significant surges in many countries such as Australia and New Zealand recently (Burnet Institute, 2022; World Health Organization, 2022).

The pandemic has exacerbated known risk factors for mental disorders in general, but perinatal mental health during and in the aftermath of COVID-19 is of particular concern (Hermann et al., 2021; Shuffrey et al., 2022). Perinatal mental disorders occur during pregnancy and in the first year after giving birth, and encompass a wide spectrum ranging from mild anxiety and depression to psychosis, and including new episodes of pre-existing mental disorders (Howard and Khalifeh, 2020). They are among the commonest morbidities of pregnancy and are associated with considerable maternal and foetal/infant morbidity and mortality (Howard et al., 2014; Jones et al., 2014), and a significant cost burden, particularly to health and social care (Howard and Khalifeh, 2020). Perinatal mental disorders increase mortality and morbidity from causes such as suicide or substance misuse but are also associated with increased risks of adverse physical outcomes, such as preterm births and foetal growth impairments, while those with severe mental illness also have increased risks of pre-eclampsia, haemorrhage, placental abruption and stillbirth (Howard and Khalifeh, 2020). However, despite this, they remain underdiagnosed and undertreated (Faisal-Cury et al., 2021; Vesga-Lopez et al., 2008), and mental health care is accessed by only a small proportion of women with perinatal mental disorders (Byatt et al., 2016).

The perinatal period is already one of significant biological, psychological and social change, and before COVID-19, perinatal mental health conditions of different types and severity were estimated to affect around 10–20% of pregnancies (Stein et al., 2014). A number of studies across different methodologies and countries have reported increased rates of perinatal anxiety and depressive symptoms and disorders during the pandemic (e.g. Chmielewska et al., 2021; Fan et al., 2021; Hessami et al., 2020; Iyengar et al., 2021; Kotlar et al., 2021; Sun et al., 2020; Suwalska et al., 2021; Tomfohr-Madsen et al., 2021; Vigod et al., 2021; Yan et al., 2020). There are a number of factors which may have contributed to the increased risk for perinatal mental disorders during COVID-19. Public health measures including quarantine, physical distancing, closure of schools and social services have exacerbated known risk factors for maternal mental disorders such as financial strain, reduced psychosocial support and exposure to domestic violence (Hermann et al., 2021). In addition, direct effects of SARS-CoV-2 infection may also contribute to increased rates; infection is associated with increased rates of neurological and psychiatric outcomes in the general population, especially in those with more severe infection (Taquet et al., 2021), and a number of case reports have suggested a possible increase in postpartum psychoses after infection, although numbers are very small (Bider and Coker, 2021).

There have also been significant disruptions to mental health services. Shortages of staff existed before the pandemic and have continued. Mother and baby units (MBUs) and specialist mental health beds have faced particular challenges: length of stay has been shorter with greater use of compulsory admissions, a higher proportion of psychotic illness and overall acuity (Cranshaw et al., 2021), and adaptations have been needed to manage reduced numbers of beds, strict infection control and isolation measures and the need to implement individual COVID-19 management and discharge plans (https://oxfordhealthbrc.nihr.ac.uk/our-work/oxppl/pregnancy-and-the-perinatal-period/).

Even when available, women and clinicians may have been hesitant to access mental health emergency services and inpatient stays when needed. This has meant that more severe illness has been managed as an outpatient and often remotely (Hermann et al., 2021). Mental health support has also been restricted in general obstetric services (for example, by restrictions on partners and visitors, use of PPE and reduced length of hospitalisation post-delivery limiting opportunities for support and planning for mental health management). Both types of service have had some replacement by telehealth, and while working well for many women, those with increased vulnerability to mental illness or high-risk behaviour, domestic violence and social deprivation may require additional or alternative resources (Hermann et al., 2021; Wilson et al., 2021).

In addition to altered services, pregnant women have been asked to, or chosen to, practice more extreme social distancing during the pandemic (Hermann et al., 2021). This is not without reason: while pregnant women have a similar risk of contracting SARS-CoV-2 infection to the background population, the risks of developing severe disease, admission to the intensive care unit and mortality are all increased, particularly if the infection is contracted in the third trimester of pregnancy (Nana et al., 2022). Pregnant women with COVID-19 are also more likely to deliver preterm, and their babies are more likely to be admitted to the neonatal intensive care unit (Allotey et al., 2022). While the restrictions are understandable, they inadvertently conflict with the usual guidance for behavioural management in depression and anxiety and also result in reduced opportunities for standard care and for enhanced monitoring for at-risk patients.

The impact of the pandemic also needs to be evaluated not only in terms of the mental health of the mother but also in potential effects on the neurodevelopment of babies, and the relationship between mother and baby. There is a lack of definitive evidence to support direct vertical transmission of SARS-CoV-2 or direct effects of in utero viral exposure which might directly affect neurodevelopment, but there is some early evidence that maternal psychological experiences and increased stress during pregnancy in the context of the pandemic could influence foetal development and functioning (Firestein et al., 2022; Kokkinaki and Hatzidaki, 2022). For women in the perinatal period, general pandemic-related stresses are associated with concerns about access to support in pregnancy and afterwards, the potential impact of COVID-19 in pregnancy and newborns, and the emotional elements of the transition to parenthood (Bogler et al., 2021; Preis et al., 2020). These are in addition to specific stresses associated with perinatal mental disorders when these are present. Stress-based changes in postpartum maternal behaviour and mother-infant bonding may affect child outcomes (Bind et al., 2021; Firestein et al., 2022). In the mother-infant dyad, maternal behaviour promotes reciprocal behaviours in the infant, which in turn elicit or reinforce different parenting approaches. The pandemic’s impact on these interactions and the overall well-being of the relationship can come from multiple routes. For example, poorer mother–infant bonding was associated with higher maternal depression and grief during the COVID-19 pandemic (Liu et al., 2022; Viaux-Savelon et al., 2022), higher perinatal maternal anxiety at delivery (Provenzi et al., 2021), delivery during the pandemic in general (Mayopoulos et al., 2021) and days of separation in infants to mothers with a SARS-CoV-2 infection (Wang et al., 2020).

Clinicians and patients in perinatal mental health have been faced, therefore, with particular challenges in addressing these multifactorial risks to mother and child in the perinatal period, during and in the aftermath of the COVID-19 pandemic. Areas of uncertainty such as how to organise specialist services, when and how to use virtual or in-person consultation and the assessment of risk particularly in women with multiple mental, physical and social risk factors are key issues in this area.

These extra challenges have been on the background of significant variations in perinatal mental health services both between and within countries and regions, which existed even pre-pandemic (Howard and Khalifeh, 2020). General medical services are key in the early identification of perinatal mental disorders, but vary by country and region. Specialist mental health care is accessed by only a small proportion of women with perinatal mental disorders (Byatt et al., 2019) and even when available, services such as specialist outpatient clinics and MBUs tend to cluster around major cities or urban areas, with geographical inequity of access (Howard et al., 2022a). In addition, not all countries or areas within countries have experienced the same restrictions and stressors as a consequence of the pandemic. For example, in Australia, national containment measures were used initially, but the response to a second wave of COVID-19 infections was more localised. Melbourne entered an additional lockdown, one of the longest and most stringent pandemic lockdowns in the world at the time (Hui et al., 2021; *The Washington Post*, 2020). While effective in containing the spread of the virus, these measures and local variations have significant economic, social and personal impacts including on perinatal mental health (Lequertier et al., 2022).

As the pandemic has evolved from an acute crisis and beyond, clinicians, patients, and health care organisations have needed access to reliable and pragmatic clinical guidance and evidence (Smith et al., 2020a). In the initial phase of the pandemic, there were frequent updates and guidelines from specialties, countries and world organisations, and while the amount of information available for the busy clinician could be overwhelming, it was not always relevant to the specific concerns of specialist mental health services. As we are learning to live with the virus, clinicians will continue to need guidance on navigating a more hybrid and adaptable approach to management.

As mental health clinicians, we assessed available guidance on the management of perinatal mental disorders during the COVID-19 pandemic using a validated evidence-based approach (Smith et al., 2020a): a team of researchers with multidisciplinary backgrounds (including mental health clinicians, researchers, methodologists, and a pharmacist) systematically searched English language websites for guidelines on perinatal mental health in the context of the current COVID-19 pandemic and afterwards (updated June 2022). References on each website were also searched and supplemented with Google searches using keywords relevant to COVID-19, perinatal mental health and guidelines. Queries or disagreements were resolved by team discussion, and the team collaborated with experts in the field to keep the guidance global, focused and comprehensive. The results of the search and synthesis of guidelines on perinatal mental health are available at https://oxfordhealthbrc.nihr.ac.uk/our-work/oxppl/pregnancy-and-the-perinatal-period/ and show that, in general, the COVID-19 guidance available for perinatal mental health services is relatively sparse, focused mainly on obstetric care and almost entirely from a small number of sources and countries. Much of the guidance is UK focussed (despite a broad and comprehensive search, which included also the United States, Australia, New Zealand, Canada and Singapore) and therefore not always applicable to other settings.

In particular, the use of telepsychiatry in perinatal mental health, particularly after the acute phase of the pandemic, is not specifically addressed in the current guidance. This is an area where clinicians may need to navigate a hybrid approach, and formal guidance would be helpful. Prior to the pandemic, there was already a strong evidence base for general telepsychiatry (American Psychiatric Association, 2022), with examples of added value in bringing together subspecialty expertise and benefits in terms of efficiency and privacy. While further targeted research is needed in telepsychiatry for perinatal mental disorders (Vigod and Dennis, 2020), there is already strong evidence of effectiveness and acceptability across other settings and disciplines in psychiatry and across different cultures (American Psychiatric Association, 2022). Living with COVID-19 may mean a variable and flexible response by services to rising and falling rates of infection. In addition, telepsychiatry itself offers some benefits over in person meetings, and so the learning and experience of its use should not be abandoned as we move forward after the acute crisis. To integrate telepsychiatry successfully into the post-COVID-19 plan for clinical practice, clinicians, patients and health care organisations need access to reliable and pragmatic clinical guidance and evidence. Evidence-based summaries of guidance on telepsychiatry in the context of COVID-19 are available (Smith et al., 2020b), but there is an absence of formal guidance specifically for perinatal mental health care.

Telepsychiatry had already been investigated pre-pandemic as a possible route for increasing access and cost effectiveness in perinatal services (Shore et al., 2020). It offers some specific advantages in this setting including increased accessibility to specialised perinatal mental health care, which tends to be concentrated in large urban cities and academic centres. Telepsychiatry may therefore particularly benefit perinatal women in rural areas who are known to be at a higher risk for anxiety or depression (Ginja et al., 2020). It also offers the possibility of a more naturalistic assessment of the caregiver–infant interaction, evaluation of the whole family including other caregivers and siblings and easier collaboration with the extended multidisciplinary team (Gressier et al., 2021). Pre-COVID-19, a systematic review of Internet-delivered psychological interventions for clinical anxiety and depression in perinatal women found improvements in depression and anxiety symptoms (Loughnan et al., 2019). A systematic review and meta-analysis on the efficacy of tele-interventions for the perinatal population also found a reduction in depressive and anxiety symptoms in women with postpartum depression (Zhao et al., 2021). Patients within perinatal services also reported high levels of satisfaction with use of telemental health interventions (Ackerman et al., 2021).

The pandemic accelerated the adoption of telepsychiatry in perinatal care and increased patients’ and clinicians’ confidence in using the various platforms and media available, but treatment still needs to be adapted to the needs of the individual and not all interventions are the same. Small differences in design, delivery and frequency of contact can affect acceptability, adherence and outcome. For example, adherence to a web-based psychological treatment was higher with telephone-based coaching than with low-intensity online support (Loughnan et al., 2019). In addition, the well-documented ‘digital divide’ is also especially relevant in perinatal mental health care: future research should target virtual care approaches that improve access among socio-economically vulnerable populations, those with limited access to Internet, video platforms or telephone, and those who have difficulty finding a private safe space for virtual meetings (Vigod and Dennis, 2020). Access to video is particularly important in perinatal mental health (to assess the parent–infant interaction and evaluation of social and family support), but may not be available. Many patients may feel unable to access digital health because of their lack of skill or confidence. Training programmes for patients (Hoffman et al., 2020) have been effective in increasing patients’ confidence and competence, but practical access to relevant technology remains a significant issue. Finally, effectiveness and acceptability across different cultures and backgrounds is key area, and standard interventions may require targeted modification.

Not only do patients vary in their skills and confidence in accessing digital health, so do clinicians. The pandemic has accelerated the use of telepsychiatry in general, but pre-COVID concerns from mental health clinicians including lacking detailed knowledge of the wider range of telepsychiatry techniques, experience in establishing rapport and therapeutic alliance (Torous and Wykes, 2020), and key areas of skills such as assessing risk and safeguarding (Cowan, 2019) have not yet been fully addressed. Training clinicians will be a key area, with an evidence-based, focused and measurable training approach to meet the needs of a post-COVID-19 world (Smith et al., 2022). In perinatal mental health services, there are extra challenges in assessment and delivery of interventions remotely, but there are also already examples of successful adaptations, for example, in delivering infant–parent therapy via telehealth (Ehmer et al., 2022; Paul et al., 2022).

Assessment of risk is a priority in perinatal mental health services, and this needs to be managed safely, whether in person or remotely. There is evidence that rates of reporting of domestic violence and abuse (DVA) fell during the pandemic (Hildersley et al., 2022), and this may be related to the difficulty for some women of safely disclosing DVA across a virtual interaction when they are at home with the perpetrator. There is a need to guide professionals in how to screen for and assess risk both remotely and safely (Emezue, 2020; Howard et al., 2022b). Several organisations have produced guidance on assessing risk remotely (see https://oxfordhealthbrc.nihr.ac.uk/our-work/oxppl/domestic-violence-and-abuse/ for summary). In addition, some new emergency support routes which were developed and used during the pandemic, such as using existing public places and increases in pre-existing support (helplines and shelters), can be used in addition to remote assessments. Techniques such as the use of code words or signals during a consultation may also be helpful. However, for some patients who are at higher risk of DVA or self-harm, or relapse of severe mental illness, in-person meetings will be essential to assess these risks. For others, a hybrid model of telemedicine and in-person meetings may be the optimal solution (Wassef and Wassef, 2022).

In summary, perinatal services, consistent with all health care services, rapidly shifted during the pandemic to the use of telepsychiatry. Given the clear advantages, we should not lose the impetus to integrate telepsychiatry into practice in the post-COVID-19 world. Mental health services should explore ways to use technology to improve integration of services and facilitate access to perinatal mental health treatment. Mobile applications are particularly accessible and provide low-cost opportunities. With the increasing access to smartphones, there is the potential to address some of the urgent challenges in ensuring prompt and equitable access and uptake for perinatal health services globally. Rigorously designed trials are needed to address some of the unanswered questions in this specialist setting (Vigod and Dennis, 2020) and approaches will need to be individualised according to the needs and risks of the patient.

Aside from the use of telepsychiatry, there are wider issues in perinatal care highlighted by the pandemic which clinicians and managers will need to consider and where formal guidance would be helpful (Otu and Yaya, 2022). The pandemic and its aftermath are still evolving, with different infection rates, restrictions and vaccination rates according to region and country. This needs to be carefully considered in care planning. Clinicians and patients should agree plans for preventive care and immediate treatment of emerging symptoms, including the potential for unexpected events, particularly separation from usual sources of support. The plan should include patients and their families in monitoring symptoms, with a safety plan of response consistent with pandemic-specific restrictions at the time (Hermann et al., 2021). The longer-term impacts of the pandemic also need to be considered in service planning. Alongside the normal experience of parenting stress following the transition to parenthood, new parents are now subject to additional anxieties in the context of the COVID-19 pandemic. Parenting stress is associated with longer-term negative impacts on the parenting provided as well as on infant outcomes, but can be reduced by greater parental support (Brown et al., 2020; Taubman-Ben-Ari et al., 2021).

Women with a history of depression or anxiety disorders should continue to use preventive psychotherapies. These may be in person or via virtual health platforms according to clinical need and patient preference (Hermann et al., 2021). Psychotherapy is often effective in depression of mild and moderate severity, but antidepressants may be required in more severe depression or when therapy does not lead to remission. This is already a complex decision for patients and clinicians in balancing potential benefits and harms in each individual situation (Brown and Vigod, 2021) and will need to also include the additional risks of untreated or undertreated illness during pandemic and post-pandemic situations.

Many professional organisations and treatment providers have developed psychoeducational materials for patients and carers (see https://oxfordhealthbrc.nihr.ac.uk/our-work/oxppl/pregnancy-and-the-perinatal-period/ for examples), and voluntary and community organisations provide vital maternal mental health support as a key addition to formal mental health services. However, strategies are required on all levels, and there need to be system-level changes to address not only the pre-existing deficiencies in care provision in perinatal mental health, but also the extra pressures resulting from the pandemic. While clinicians can make a significant impact by focussing on prevention, treatment, risk assessment, and symptom monitoring, the task of organising perinatal mental health services for the current ongoing crisis and preparing for the next needs guidance and planning across organisations including government departments, health care providers, and the voluntary sector.
